# An expanded role for heterozygous mutations of *ABCB4, ABCB11, ATP8B1, ABCC2* and *TJP2* in intrahepatic cholestasis of pregnancy

**DOI:** 10.1038/s41598-017-11626-x

**Published:** 2017-09-18

**Authors:** Peter H. Dixon, Melissa Sambrotta, Jennifer Chambers, Pamela Taylor-Harris, Argyro Syngelaki, Kypros Nicolaides, A. S. Knisely, Richard J. Thompson, Catherine Williamson

**Affiliations:** 1 Division of Women’s Health, King’s College London, London, UK; 2 Division of Transplantation Immunology & Mucosal Biology, Liver Sciences, King’s College London, London, UK; 3 Harris Birthright Centre for Fetal Medicine, King’s College Hospital, London, UK; 4 Institute of Liver Studies, King’s College Hospital, London, UK; 5 Present Address: Institut für Pathologie, Medizinische Universität Graz, Graz, Austria

## Abstract

Intrahepatic cholestasis of pregnancy (ICP) affects 1/140 UK pregnancies; with pruritus, hepatic impairment and elevated serum bile acids. Severe disease is complicated by spontaneous preterm delivery and stillbirth. Previous studies have reported mutations in hepatocellular transporters (*ABCB4*, *ABCB11*). High throughput sequencing in 147 patients was performed in the transporters *ABCB4*, *ABCB11*, *ATP8B1*, *ABCC2* and tight junction protein 2 (*TJP2*). Twenty-six potentially damaging variants were identified with the following predicted protein changes: Twelve *ABCB4* mutations - Arg47Gln, Met113Val, Glu161Gly, Thr175Ala, Glu528Glyfs*6, Arg590Gln, Ala601Ser, Glu884Ter, Gly722Ala, Tyr775Met (x2), Trp854Ter. Four potential *ABCB11* mutations - Glu297Gly (x3) and a donor splice site mutation (intron 19). Five potential *ATP8B1* mutations - Asn45Thr (x3), and two others, Glu114Gln and Lys203Glu. Two *ABCC2* mutations - Glu1352Ala and a duplication (exons 24 and 25). Three potential mutations were identified in *TJP2*; Thr62Met (x2) and Thr626Ser. No patient harboured more than one mutation. All were heterozygous. An additional 545 cases were screened for the potential recurrent mutations of *ATP8B1* (Asn45Thr) and *TJP2* (Thr62Met) identifying three further occurrences of Asn45Thr. This study has expanded known mutations in *ABCB4* and *ABCB11* and identified roles in ICP for mutations in *ATP8B1* and *ABCC2*. Possible novel mutations in *TJP2* were also discovered.

## Introduction

Intrahepatic cholestasis of pregnancy (ICP), also called obstetric cholestasis, is the commonest pregnancy-specific liver disease. Typically presenting in the third trimester, ICP can be debilitating for the mother and threaten the health of the fetus^1–3^. Affected women have abnormal values of hepatobiliary-injury biomarkers, including raised serum bile acid (BA) concentrations^4^, that typically resolve by 12 weeks postpartum. Adverse pregnancy outcomes occur more commonly when maternal serum bile acid concentrations are >40 µmol/L. They include spontaneous preterm labour, prolonged admission to the neonatal intensive care unit and third trimester intrauterine death^2,3,5,6^. ICP is associated with a disrupted metabolic profile^7,8^ and can have long-term consequences for the health of the mother^9,10^ and child^11^.

The aetiology of ICP is complex and not fully understood, but a role has been established for the effect of elevated reproductive hormones towards the end of pregnancy (estrogens and sulphated progesterone metabolites), when the disease most commonly presents (^12,13^, reviewed in ref.^14^). Indications of genetic involvement in disease susceptibility are clear (reviewed in ref.^15^). Pedigree studies have identified families where ICP is inherited in an autosomal dominant sex-limited fashion^16,17^. The varying geographical prevalence also indicates genetic involvement, with population-specific genetic backgrounds conferring different levels of risk^18^. Historically the prevalence of ICP was highest amongst the Native Chilean population^19^. Further evidence of genetic involvement comes from sibling studies that show a 12-fold increase in risk between parous sisters^20,21^.

Severe childhood liver disease can be caused by homozygous mutations in genes that encode transporters involved in bile formation at the hepatocyte canalicular membrane. These transporters play roles in cholestatic disorders that range in severity from progressive paediatric disease to transient cholestasis associated with drug ingestion or pregnancy (ICP)^22^.

Studies of the gene *ABCB4*, (ATP binding cassette subfamily B member 4) encoding the phosphotidylcholine floppase MDR3 and homozygously mutated in progressive familial intrahepatic cholestasis (PFIC) type 3, identified a heterozygous mutation of *ABCB4* first in a familial case^23^ and then in a sporadic instance of ICP^24^. Subsequent studies have identified a wide range of mutations of *ABCB4* in ICP, most commonly missense mutations^15^. Approximately 10% of women with ICP harbour a mutated allele of *ABCB4* ^15^. Mutations of *ABCB4* have also been implicated in drug-induced cholestasis^25^ and low phospholipid-associated cholelithiasis^26^.

The bile salt export pump (BSEP), encoded by the gene *ABCB11*, (ATP binding cassette subfamily B member 11) is homozygously mutated in PFIC type 2 and benign recurrent intrahepatic cholestasis (BRIC) type 2^27^. Several studies found *ABCB11* mutations in women affected with ICP^28–30^.

Of note is that in addition to causative mutations of *ABCB4* and *ABCB11* acting as Mendelian-like alleles, population studies of ICP cohorts have identified common genetic variants at these loci that contribute to disease risk, albeit to a much smaller degree than causative mutations^31^.

The third PFIC (and BRIC) gene, *ATP8B1*, (ATPase class 8B member 1) encoding a phosphatidyl serine flippase (familial intrahepatic cholestasis 1, FIC1), has only been studied to a limited extent (with respect to sequencing) in ICP cohorts, and hence the role of genetic variation at this locus in ICP is not established^32,33^. A functional interdependence of this flippase with MDR3 in hepatocytes suggests that *ATP8B1* remains a viable candidate for involvement in ICP susceptibility^34^, as does the link to ABCB11 function through FXR/PLD2 signalling^35^.

Another canalicular ATP-driven transporter, the multidrug resistance-associated protein 2 (MRP2, encoded by the gene *ABCC2*(ATP binding cassette subfamily C member 2) is involved in bile formation. This protein exports bilirubin, some bile acids and many other anion conjugates into bile. Homozygous mutations of *ABCC2* cause the rare liver disorder Dubin-Johnson syndrome, which presents with conjugated hyperbilirubinaemia^36^. Although common variation and ICP susceptibility have been investigated at this locus in population cohorts, with conflicting results^31,37,38^, there are no published analyses of comprehensive sequencing of the coding region of *ABCC2* in an ICP cohort.

Familial forms of severe progressive cholestasis without a confirmed genetic diagnosis also exist^39^. Mutations of another gene, tight junction protein 2 (*TJP2*) have recently been implicated in some patients with such disease^40^. The encoded protein named zona occludens 2 (ZO-2) is a cytosolic component of a number of types of cell-cell junctions at many sites, interacting with membrane proteins and cytoskeletal proteins. The detergent actions of bile acids in the liver on mutated cellular junctions are postulated to cause the cholestatic phenotype^40^. To date this gene has not been studied in ICP. Given the role of other PFIC genes in ICP, *TJP2* seems a reasonable candidate for analysis.

To clarify the extent of involvement in ICP of these genes implicated in familial cholestasis we undertook high-throughput targeted sequencing of *ABCB4, ABCB11, ATP8B1, ABCC2* and *TJP2* in a cohort of 147 women with ICP.

### Patients

The study included 147 women with a confirmed diagnosis of ICP, recruited through the Women’s Health Research Centre at Queen Charlotte’s and Chelsea Hospital, Imperial College Healthcare NHS trust, St. Mary’s Hospital Imperial College London, and North Lincolnshire and Goole Hospitals NHS foundation trust. All patients provided written informed consent, conforming to the 1975 Helsinki guidelines; permission for the study was granted by the ethics committee of the Hammersmith Hospitals NHS Trust, London (references REC97/5197 and 08/H0707/21).

ICP was diagnosed as described previously on the basis of clinical symptoms (pruritus without identifiable skin lesions other than excoriations) and routine laboratory investigations^33^. ICP was confirmed with detection of raised maternal serum bile acids. Women were excluded if viral or autoimmune hepatobiliary disease was diagnosed. A larger cohort of 545 patients with ICP, described in a previous study^31^, was used to evaluate potential recurrent mutations.

## Results

Genes implicated in familial cholestasis underwent targeted next-generation sequencing using the MiSeq platform in a cohort of 147 ICP patients. This identified 26 potentially pathogenic variations. Table 1 shows each variant identified together with its predicted protein consequence. Clinical findings for each patient in whom a potential mutation was identified are also shown (Table 2). All of the described mutations were observed in the heterozygous state and no patient harboured more than one potentially pathogenic variant. The frequency of two of the previously identified common susceptibility variants for ICP^31^; namely rs2109505 in *ABCB4* and rs2287622 in *ABCB11*, was noted in these individuals (Table 1). All of the missense variants identified, except where specified (Table 1) were predicted to be pathogenic by SIFT and MutationTaster.

**Table 1:** Potential pathogenic variants identified in this study.

	**Patient**	**DNA Change**	**Predicted Protein Change**	**Prediction pathogenic?**	**Database ref**	**rs2287622**	**rs2109505**
***ABCB4***	1	c.140G > A	p.Arg47Gln	y	rs372685632	VA	AT
	2	c.337A > G	p.Met113Val	y	rs757245592	VA	AA
	3	c.482A > G	p.Glu161Gly	y	NV	VA	AT
	4	c.523A > G	p.Thr175Ala	y	rs58238559	VA	AA
	5	c.1583delA	p.glu528glyfs*6	y	NV	VA	TT
	6	c.1769G > A	p.Arg590Gln	y	rs45575636	VV	AT
	7	c.1801G > T	p.Ala601Ser	y	NV	AA	AA
	8	c.2050G > T	p.Glu684Ter	y	NV	VV	AA
	9	c.2165G > C	p.Gly722Ala	y	rs779885518	VV	AT
	10	c.2324C > T	p.Tyr775Met	y	rs148052192	VA	AA
	11	c.2324C > T	p.Tyr775Met	y	rs148052192	AA	AA
	12	c.2561G > A	p.Trp854Ter	y	NV	VV	AA
***ABCB11***	13	c.890A > G	p.Glu297Gly	y	rs11568372	AA	AA
	14	c.890A > G	p.Glu297Gly	y	rs11568372	VA	AA
	15	c.890A > G	p.Glu297Gly	y	rs11568372	AA	AT
	16	c.2343 + 1G > T	Intron 19 splice	y	rs774411820	AA	AA
***ATP8B1***	17	c.134A > C	p.Asn45Thr	y	rs146599962	AA	AA
	18	c.134A > C	p.Asn45Thr	y	rs146599962	VV	AA
	19	c.134A > C	p.Asn45Thr	y	rs146599962	VA	AA
	20	c.340G > C	p.Glu114Gln	y	rs753142591	VA	AT
	21	c.607A > G	p.Lys203Glu	y	rs56355310	VA	AT
	27	*c.134A* > *C*	*p.Asn45Thr*	*y*	*rs146599962*	*NT*	*NT*
	28	*c.134A* > *C*	*p.Asn45Thr*	*y*	*rs146599962*	*NT*	*NT*
	29	*c.134A* > *C*	*p.Asn45Thr*	*y*	*rs146599962*	*NT*	*NT*
***ABCC2***	22	c.4055A > C	p.Glu1352Ala	y	NV	AA	AT
	23	dupl. ex 24–25	ins then Ter	1	esv3423829	AA	AA
***TJP2***	24	c.185C > T	p.Thr62Met	y/n 2	rs138241615	AA	AA
	25	c.185C > T	p.Thr62Met	y/n 2	rs138241615	VA	AA
	26	c.1877C > G	p.Thr626Ser	y/n 2	rs149911553	AA	AA

Notes: Individuals in italics were identified by second screen of larger cohort.

NV - novel variant not currently reported in dbSNP.

NT - not typed.

1 Insertion is out of frame.

2 SIFT predicts tolerated, mutation taster predicts disease causing.

rs2287622 shows amino acids at this SNP, rs2109505 is nucleotides (SNP is synonymous).

**Table 2:** Clinical and biochemical findings in the individuals with potential mutations.

	Patient	ALT	ALP	GGT	Bilirubin	Bile Acids	Maternal Biliary Disease	F/H Biliary Disease	Adverse pregnancy outcomes
***ABCB4***	1	345	159	20	40	**150**	**no**	**no**	SPL, M
	2	27	204	13	4	**16**	**no**	**no**	PPH
	3	45	280	18	12	**39**	**no**	**no**	no
	4	35	284	NP	6	**30**	**no**	**no**	no
	5	172	522	20	13	**239**	**G**	**G, ICP**	no
	6	291	244	18	8	**39**	**G**	**G**	no
	7	1802	710	10	18	**108**	**no**	ICP*	M, FHR
	8	58	261	NP	NP	**78**	**no**	**G**	no
	9	579	270	NP	NP	**112**	**G**	**G**	M, FHR
	10	115	376	28	7	**44**	**no**	**Cirr**	no
	11	776	292	NP	57	**50**	**G, C**	**G, ICP**	FHR
	12	803	204	66	NP	**222**	**no**	**G, Cirr**	no
***ABCB11***	13	229	238	NP	23	**118**	**G, C**	**nk**	SPL, M, FHR
	14	94	190	12	8	**23**	**no**	**no**	no
	15	NP	91	8	4	**14**	**no**	**no**	no
	16	32	302	11	4	**44**	**G**	**no**	NNU
***ATP8B1***	17	410	344	29	16	**27**	**no**	**no**	PPH
	18	131	247	38	26	**69**	**no**	**no**	no
	19	707	263	17	20	**120**	**G**	**G**	FHR
	20	23	227	36	10	**35**	**G**	**G, Cirr**	no
	21	96	261	33	9	**22**	**no**	**no**	no
	*27*	*506*	*510*	*110*	*16*	***182***	***no***	***G***	*SPL*
	*28*	*415*	*358*	*32*	*18*	***16***	***nk***	***nk***	*no*
	*29*	*101*	*323*	*18*	*4*	***17***	***no***	***no***	*FHR*
***ABCC2***	22	14	291	23	11	**66**	**no**	**no**	no
	23	80	257	NP	10	**73**	**no**	**G**	no
***TJP2***	24	110	178	NP	9	**57**	**nk**	**nk**	no
	25	225	435	26	17	**42**	**no**	**no**	SB
	26	740	527	NP	NP	**127**	**no**	**no**	no

Individual in italics identified by second screen of larger cohort.

NP – not performed.

G – gallstones.

PPH - post partum haemorrage.

ALT - alanine transaminase.

C - oral contraceptive induced cholestasis.

SPL - spontaneous preterm labour (<37 weeks).

ALP - alkaline phosphatase.

ICP - intrahepatic cholestasis of pregnancy.

SB - still birth in previous pregnancy.

GGT - gamma glutamyl transferase.

Cirr - liver cirrhosis.

M - mecconium staining.

F/H - family history.

FHR - fetal heart rate abnormalities.

nk - not known.

NNU - > 48 hr admission to neonatal unit.

*- based on recollection of maternal aunt.

### *ABCB4* variants

Consistent with previous findings^15^, the largest number of potential mutations (11 different variants in 12 patients) were identified in the gene encoding the phosphatidyl choline floppase *ABCB4* in this patient cohort (Table 1). Two predicted resulting variants, namely Thr175Ala and Arg590Gln, have previously been reported as pathogenic in cholestatic disease^41,42^. Three novel mutations are predicted to result in a frameshift leading to a stop codon six missense amino acids thereafter (Glu528Glyfs*6) and two immediate introductions of a premature stop codon (Glu684Ter, Trp854Ter), a type of mutation only very infrequently previously described in ICP^15^. The remaining 7 mutations are novel missense changes predicted to be pathogenic (Alamut).

### ABCB11 variants

Fewer potentially pathogenic variants (n = 2, 4 patients) were identified in *ABCB11*, encoding the bile salt export pump. A recurrent mutation, predicted to yield the substitution Glu297Gly, first described in PFIC2 patients^27^, and later identified in a study of ICP patients^29^, was discovered in 3 separate patients not known to be related. A donor splice site mutation in intron 19 was identified with the predicted consequence of an exon skipping event. A missense variation, resulting in the protein change Asn591Ser (dbSNP: rs11568367), was identified in an additional patient, which has previously been reported in ICP^28^. However, despite this amino acid being highly conserved, bioinformatic tools predict the variation to be benign; this allele is also relatively common in the South Asian population (MAF 11%). Thus, it seems not likely to be a pathogenic variant.

### ATP8B1 variants

Several variants of interest were identified in *ATP8B1*, encoding a phosphatidyl serine flippase. A mutation predicted to be pathogenic by SIFT and Mutation Taster, causing the protein change Asn45Thr, was seen in 3 unrelated individuals. Two further variants predicted to be pathogenic were identified in 2 additional patients, resulting in the protein changes Glu114Gln and Lys203Glu. To clarify the potential pathogenic and recurrent role (in ICP) of the Asn45Thr variant a cohort of 545 cases was screened for this change (see methods above); an additional 3 patients harbouring this variant were identified (Tables 1 and 2).

### ABCC2 variants

Two significant alterations were identified in *ABCC2*, encoding MRP2, a transporter of conjugated bilirubin and other organic anions. A single missense substitution, predicted to result in the protein change Glu1352Ala was identified in one patient. Alamut analysis indicated that SIFT and mutationTaster both predict the change to be deleterious. A second identified DNA change, confirmed by RT-PCR dosage methodology, was a genomic duplication of 5299 base pairs, encompassing exons 24 and 25 of the gene and predicted to result in the insertion of a premature stop codon. This copy number variant has been reported once before (database reference esv3423829).

### TJP2 variants

Three patients harboured TJP2 variants of potential interest. Two unrelated individuals had the same DNA mutation causing the protein change Thr62Met. This alteration is predicted to be disease causing by Mutation Taster; SIFT, however, predicts this change to be tolerated. A second missense alteration seen in one patient is predicted to cause the protein alteration Thr626Ser. This change is also assessed as tolerated by SIFT but as disease causing by Mutation Taster. 545 ICP cases (as above) were subsequently screened for the possible recurrent Thr62Met variant, however no further ICP cases carrying this change were identified.

### Biochemical and clinical features of ICP cases with variants

Elevations in serum BA and alanine transaminase (ALT) activity values varied among patients and there was no particular trend for greater elevation of specific analytes in women with particular mutations in specific genes. Hyperbilirubinaemia was rare. It did, however, occur in association with *ABCB4*, *ABCB11* or *ATP8B1* variants. Six women had elevated bilirubin values (>20 µmol/L); all had severe ICP (serum BA > 40 µmol/L). The two whose bilirubin concentrations were consistent with jaundice (>35 µmol/L) harboured *ABCB4* mutations. Only two women with mutations (one in *ABCB4*, one in *ATP8B1*) were known to have elevated gamma glutamyl transferase (GGT) levels. Of the 26 women with a mutation in a biliary transporter (*ABCB4*, *ABCB11*, *APT8B1*, *ABCC2*), 12 (46%) had an established family history of biliary disease (ICP, cirrhosis or gallstones).

## Discussion

This study is the largest analysis to date of the role of mutations of familial cholestasis genes in ICP susceptibility. Previous studies have identified genetic contributions to the disease primarily focussing on *ABCB4*. Here, by using a next generation sequencing approach, a panel of genes could be analysed in parallel in a large cohort of patients.

Our analysis identified a number of mutations of *ABCB4*, encoding the phosphatidyl choline floppase MDR3. *ABCB4* has been analysed in a number of ICP cohorts and mutations in this gene represent the largest overall genetic contribution to ICP susceptibility known to date^15^. Several groups have established functional assays for this protein that can incorporate mutant constructs^43–46^. Such assays help to define the impact of a given variant on protein function; bioinformatics predictors of clinical importance of variants like those used in this study are useful (and improving) but can never be conclusive. Functional assays are being used to classify mutations by mechanism of effect on protein function (*e.g*., trafficking defect resulting in mis-localisation, loss of floppase activity). This is of value in *ABCB4*-associated diseases; these data may predict response to therapy intended to rescue floppase function. It is noteworthy that in the *ABCB4* mutant carrier group, 8/12 (67%) had a family history of biliary disease (ICP, cholelithiasis or cirrhosis) (Table 2). This is consistent with a study of parous women with LPAC and co-existing *ABCB4* mutations, in which approximately 50% developed ICP when pregnant^47^.

A clear role is now established for genetic variation in *ABCB11* contributing to the aetiology of ICP, although one seemingly less than that of *ABCB4*. Our study has confirmed this role; taken together with other data, it suggests that up to 5% of ICP cases harbour a heterozygous mutation of *ABCB11* ^28–30^. In particular, the two mutant alleles found commonly in the PFIC2/BRIC2 population (Glu297Gly and Asp482Gly) are also present in the ICP population. Functional studies of *ABCB11* mutations have been performed in addition to mini-gene construct splicing analysis and immunohistochemical studies to establish genotype/phenotype correlations^48–50^; studies of this type are helpful to clarify the clinical relevance of findings of unknown significance. Two of the five women in this group had cholelithiasis (Table 2). Previous studies of ICP patients and *ABCB11* mutations have not identified this association.

Analysis of *ATP8B1* in ICP has been limited, with the largest study focussing on common SNPs and population risk rather than mutations [31–33]. In this study we confirmed a small potential role for *ATP8B1* variants to contribute to ICP. We examined a larger ICP population for the recurrent variant (Asn45Thr) and identified three further individuals harbouring it. Taken with the other mutations identified in this gene, it is therefore possible that up to 3% of ICP patients may have a heterozygous mutation of *ATP8B1*. However, the Asn45Thr variant, although predicted to be deleterious, is fairly common in the normal population (global minor allele frequency 0.49%), and has been reported in a study of chronic pancreatitis^51^. Although this does not exclude this variant from having a mildly deleterious effect on protein function, *in vitro* experimental approaches are warranted to confirm if this variant contributes to disease risk.

That our cohort included two patients with potentially pathogenic variants of *ABCC2*, a missense mutation predicted to be pathogenic and a large in-frame duplication, is of interest. No published studies report sequencing this gene in ICP; our data suggest a small but potentially overlooked contribution to ICP by heterozygous *ABCC2* mutations.

This study is the first to examine *TJP2* in an ICP cohort. We have identified some evidence for involvement of this gene, with three occurrences of variants that may predispose to disease. However, as bioinformatics prediction tools did not agree on the clinical relevance of these changes, further analysis is warranted. Development of functional assays for this protein, together with further sequencing of *TJP2* in larger patient cohorts, will be key to understanding the role of these variants.

A spectrum of variants have been identified in this and other ICP cohorts. Although some variants will be predicted to have only a mild impact upon disease susceptibility, either from database predictions or using *in vitro* assays, the cholestatic impact of gestational elevations of reproductive hormones may result in a more severe phenotype in the context of pregnancy.

Heterozygous mutations in the five genes sequenced were identified in nearly 20% of the ICP population being studied. However, the mutant-carrying groups exhibited no clear phenotypic differences (Table 2). Serum BA or ALT values did not vary by gene and raised serum GGT activity was not common in those with a mutation in *ABCB4*. Biomarkers that indicate which biliary transporter is mutated in a specific ICP patient thus remain to be found. That >50% of the women with identified variants had a family history of biliary disease is noteworthy; this information could be useful in a clinical setting to make decisions about mutation screening. Further studies with deeper phenotyping approaches (especially metabolic profiling/serum lipidomics and reproductive hormone metabolite analysis, based on our knowledge of disease pathophysiology) are warranted to determine if these mutation groups can be distinguished by clinical phenotype. Research into the impact of specific genotypes on drug response, risk of subsequent hepatobiliary disease, resolution of symptoms after delivery, duration of symptoms, and long term maternal and child health will be of particular value.

In this study, we used a candidate sequencing approach to identify mutations with a potentially large impact on protein function. Separate studies have also taken a population genetics approach using candidate single nucleotide polymorphisms and/or tagging SNPs in an attempt to identify common variants that contribute to overall risk^31,38,52,53^. Common variants that make a much smaller contribution to disease risk in ICP populations have been identified at the *ABCB4* and *ABCB11* loci (in particular rs2109505 (*ABCB4*) and rs2287622/rs7577650 (*ABCB11*)^28,31,53^). Thus, the genetic architecture of ICP is complex, with rare mutant alleles, often but not exclusively specific to each patient, present in affected women, such as reported in this study, together with common variants at the same loci that represent risk alleles as we have previously described. Although our previous study excluded a large role for common variation of *ATP8B1* and *ABCC2* in ICP^31^, studies in larger cohorts may identify variants conferring a small degree of risk (or protection), given the potential high-impact mutations of these genes identified by this study. Common variation around *TJP2* causing small alterations in risk (which this study was not powered to detect) may also play a role.

We have previously studied the role of *FXR* variants in ICP and identified a small number of rare variants with a functional effect^54^. When designing this study this gene was not included in the screening panel. However, the recent identification of *FXR* mutations in progressive familial intrahepatic cholestasis strengthen the case for further *FXR* analysis in future studies of ICP^55^.

The catalogue of ICP-associated mutations that this analysis, and others, have established permits consideration of the possibility of precision genomic medicine. With sequencing costs falling and technology continuing to advance, to contemplate screening all of the candidate genes for ICP is now feasible, enabling genetic diagnosis that in future may allow better targeted treatment and surveillance.

## Methods

Blood samples were collected in standard EDTA vacutainers and buffy coats were prepared by centrifugation using standard protocols. DNA was extracted from 200 µl of buffy coat using the Qiagen Blood mini kit (Crawley, UK). Extracted DNA was assessed for purity using an ND-1000 Nanodrop spectrophotometer (Thermofisher Scientific, Loughborough, UK) and quantified using a Qubit fluorimeter (Thermofisher).

Selected genes known or hypothesized to be involved in ICP (*ABCB4, ABCB11, ATP8B1, ABCC2* and *TJP2*) underwent targeted next-generation sequencing. Oligonucleotides (“oligos”) to screen the entire coding region of each gene were designed using the Illumina web-based sequencing assay design tool Design Studio (http://designstudio.illumina.com/) and used in the Illumina TruSeq custom amplicon v1.5 workflow according to the manufacturer’s instructions. This workflow has three main stages: 1) hybridisation of the oligos to 50 ng/µl of genomic DNA; 2) extension and ligation of the oligos; 3) PCR amplification using two unique index primers for sample. Afterwards, sample DNA concentrations were normalised using the Illumina bead-based system and pooled in a final library. The pooled library was then sequenced using a MiSeq Reagent Kit v2, 300-cycle (2 × 150bp), on the MiSeq benchtop sequencing platform (Illumina, Cambridge, UK).

Sequencing data were analysed using a bioinformatics pipeline designed in Biomedical Genomic Workbench 2.5.1 (CLC bio, Aarhus, Denmark). Sequence reads were mapped against the human reference (hg19/GRCh37). Variants were called when they had a minimum coverage of 20× and a probability of 80% of being different from the reference, in accordance with the Bayesian model incorporated into the software. Putative variants identified were filtered according to the quality and the frequency of occurrence; all changes having an allele read frequency of less than 10% and an average base quality (Phred score) of less than 20 were removed. The variants identified were further annotated using dbSNP v139 and the 1000 Genome Project database. A deeper investigation was also performed using Alamut Visual 2.7.1 (Interactive Biosoftware, Rouen, France), in which each variant was integrated with allele frequency information derived from several single nucleotide polymorphism (SNP) databases, including dbSNP v144, the 1000 Genomes Project, the Exome Aggregation Consortium and the National Heart Lung and Blood Institute Grand Opportunity Exome Sequencing Project and with *in silico* missense predictions from SIFT (sorting intolerant from tolerant) and MutationTaster. Copy number variants (CNVs) were detected via in-house dosage analysis using the read-counts generated by the NGS analysis. The CNVs were confirmed by RT-PCR using Universal Probe Library (Roche).

Each potential pathogenic variant was confirmed in patient samples using routine Sanger sequencing. Forward and reverse primers were manually designed for the flanking regions and purchased from Integrated DNA Technologies (sequences available on request).

Analysis of potential recurrent mutations of *ATP8B1* and *TJP2* were performed following primer design (“Primer picker”, LGC Genomics, Hoddesdon, UK) using a competitive allele specific PCR SNP system using fluorescence resonance energy transfer (FRET) quencher cassette oligonucleotides (KASP^TM^, LGC Genomics).

### Data Availability

Any further information on the sequencing data described in this paper is available from the corresponding author on reasonable request.
